# Triple dislocation around the knee joint: a case report

**DOI:** 10.1186/s13256-021-03201-w

**Published:** 2022-01-07

**Authors:** Ernest Chew, Aadhar Sharma, Chinmay Gupte

**Affiliations:** 1 Epsom and St Helier NHS Trust, Dorking Road, Epsom, KT18 7EG UK; 2 Imperial College Healthcare NHS Trust, South Wharf Road, Paddington, London, W2 1NY UK

**Keywords:** Knee, Trauma, Dislocation, Case report

## Abstract

**Background:**

Dislocation of the knee is a serious and potentially limb-threatening injury. There are three types of dislocation around the knee joint: patellofemoral, tibiofemoral, and tibiofibular. Tibiofemoral dislocation is the variant that is deemed the most serious, with a higher risk of compromise to the popliteal artery and common peroneal nerve. Although simultaneous dislocations of two types have been described, there has been no such description of all three types occurring simultaneously.

**Case presentation:**

We present a case of a 40-year-old hairdresser who suffered a fall off her moped in Spain, and simultaneously dislocated all three articulations around the knee. Diagnosis was achieved with clinical examination, plain films, and computed tomography and magnetic resonance imaging scans. Management consisted of initial surgical debridement and reduction with stabilization of the affected joints.

**Conclusion:**

Dislocation of the knee is an uncommon but life changing and potentially limb-threatening injury. It should always be suspected in trauma patients who present with multiligamentous knee injuries. The main concern is of neurovascular compromise to the lower leg, namely, the popliteal artery and common peroneal nerve. The treatment of multiligamentous knee injuries for most patients is surgical treatment with physiotherapy and adequate stabilization of the knee joint. Close monitoring of progress of the knee in terms of persistent laxity, range of movement, and functional status is required for at least 1-year post injury. Current evidence suggests that, despite good functional outcomes for knee dislocations in the short term, the prevalence of post-traumatic osteoarthritis is high in the long term.

## Background

The knee is a synovial joint formed by the articulations of the patella, femur, and tibia. The tibiofibular joint is a separate fibrous joint but nevertheless exists in the region of the knee and is sometimes included clinically as part of the knee.

Although all three of the patellofemoral, tibiofemoral, and tibiofibular joints can sustain dislocation, a true “knee dislocation” is defined as a complete disruption of the tibiofemoral joint with a loss of integrity of at least two of the four major knee ligaments [anterior cruciate ligament (ACL), posterior cruciate ligament (PCL), posterolateral corner (PLC), and medial collateral ligament (MCL)] [1]. This type of injury usually results from high-energy trauma. Although incidence of knee dislocation is uncommon, ranging from 0.02% to 0.2% of all musculoskeletal injuries, it is a life-changing injury when it occurs [1].

Aside from the tibiofemoral joint, patellofemoral joint dislocation is a common injury that can result from sports trauma, direct injury, or spontaneously in patients with susceptibilities such as hyperlaxity or patellofemoral dysplasia [2]. The proximal tibiofibular joint is the rarest to be dislocated, and typically results from a direct injury to the joint.

Although there have been cases of simultaneous dislocations of two of these joints in the literature [3–5], there are no reports of dislocation of all three articulations occurring simultaneously.

We report the case of a patient who was referred to our level 1 trauma center with simultaneous dislocations of the tibiofemoral, tibiofibular, and patellofemoral joints. We discuss her presentation, management, and the lessons that were learned to improve care of these patients in the future.

## Case presentation

A 40-year-old hairdresser suffered a fall off her moped in Spain after losing control at approximately 30 mph, landing onto her right side. The patient had no previous past medical or surgical history and did not take any regular medication. She was fully mobile prior to injury.

She was managed locally according to the Advanced Trauma Life Support (ATLS) guidelines. A primary survey did not reveal any life-threatening injuries that needed emergency surgery, and this was confirmed with a trauma computed tomography (CT) scan. On secondary survey, her right knee was noted to be deformed with an open wound along the medial border of the patella. The patella was also dislocated laterally and was relocated in the emergency department with adequate pain relief. The patient underwent primary surgical management at the referring center, with initial exploration and lavage of the knee joint, partial closure of the medial wound, reduction of the patella, and application of a plaster back slab, together with intravenous antibiotic treatment. The common peroneal nerve was unaffected and maintained its normal motor and sensory function throughout.

She was then transferred by air ambulance to our specialist knee trauma unit where she underwent repeat secondary survey and radiological investigations including magnetic resonance imaging (MRI) and CT.

### Investigations

**Fig. 1:**
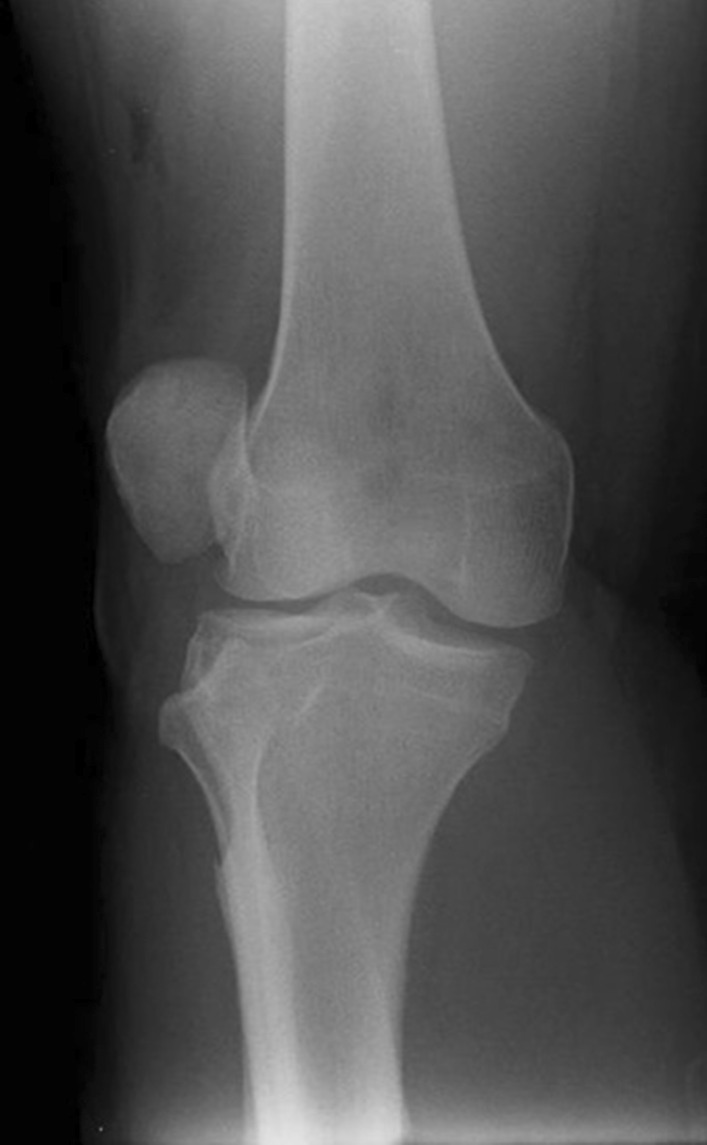
Anteroposterior (AP) view radiograph upon presentation

**Fig. 2:**
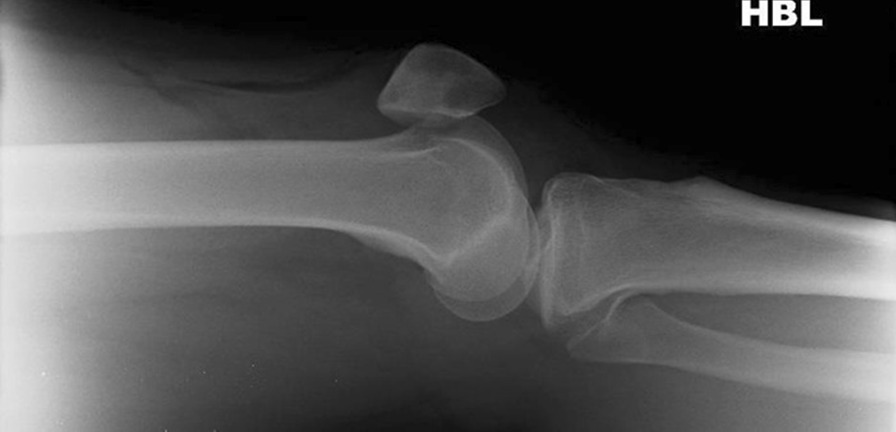
Lateral view knee radiograph upon presentation

**Fig. 3:**
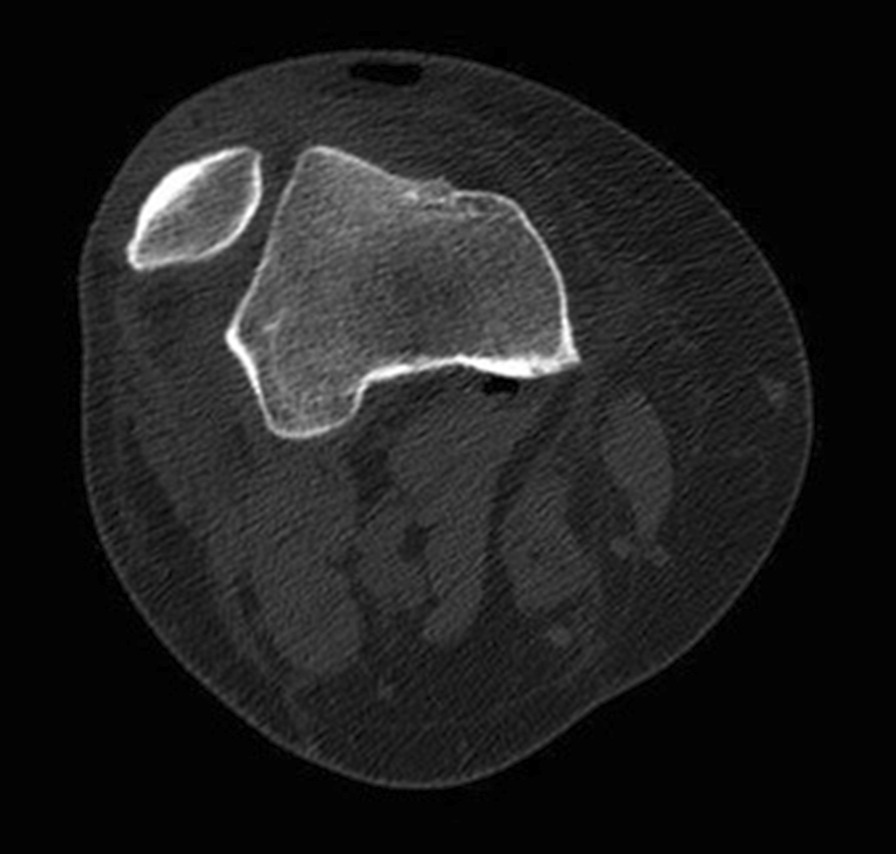
Axial view computed tomography demonstrating patellar dislocation

**Fig. 4:**
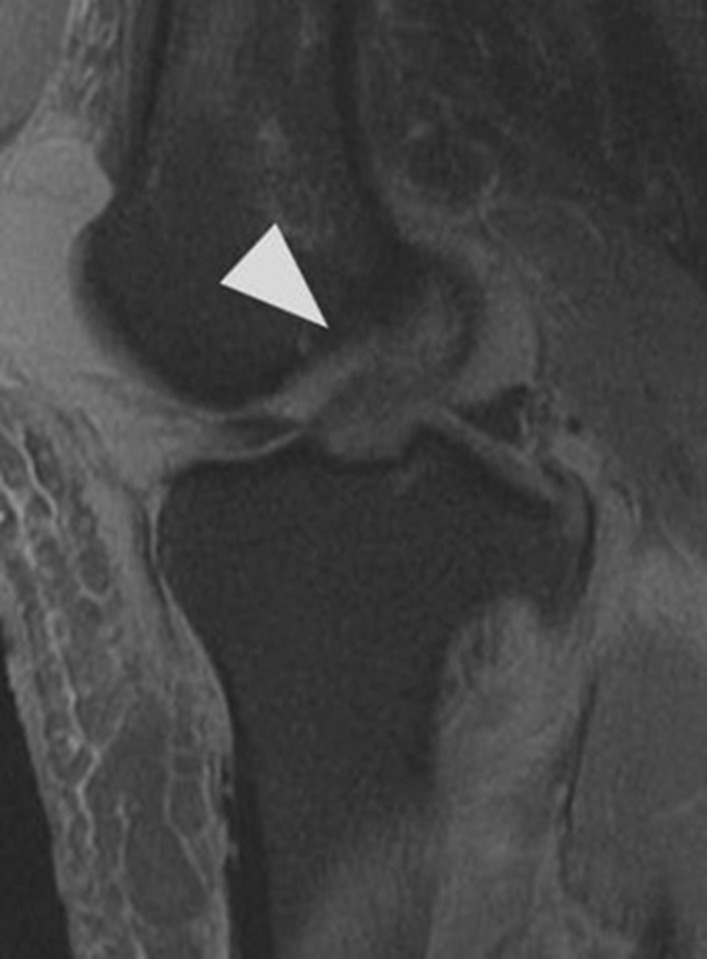
Grade 2 (partial tear) anterior cruciate ligament injury. Indicated by arrowhead

**Fig. 5:**
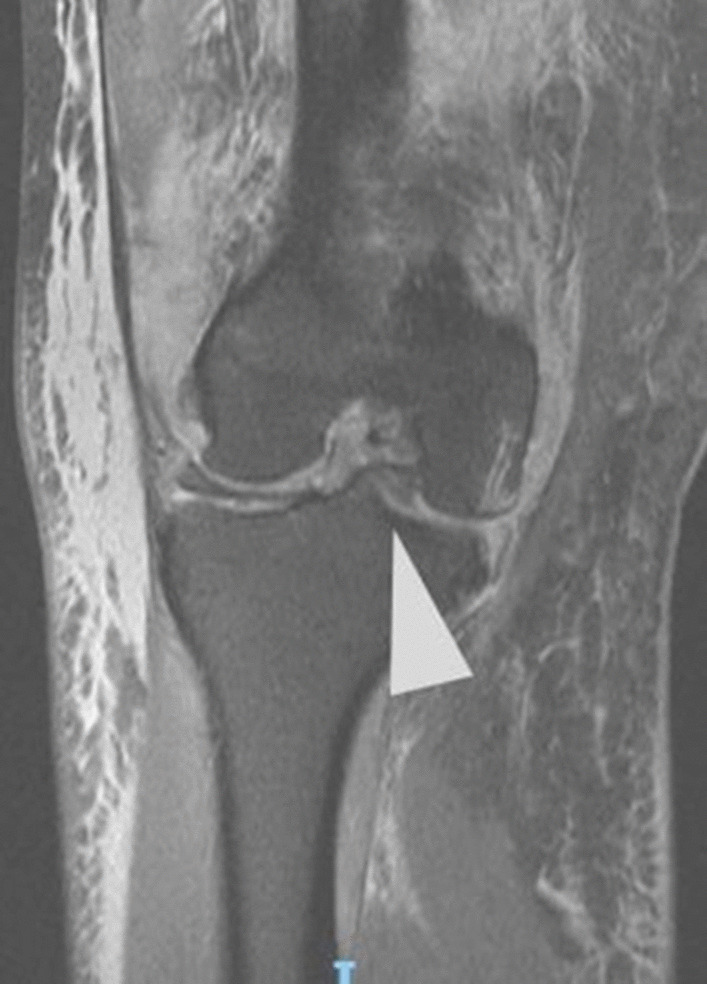
Coronal magnetic resonance imaging demonstrating bucket handle tear of medial meniscus with segment flipped into intercondylar notch, indicated by arrowhead

**Fig. 6:**
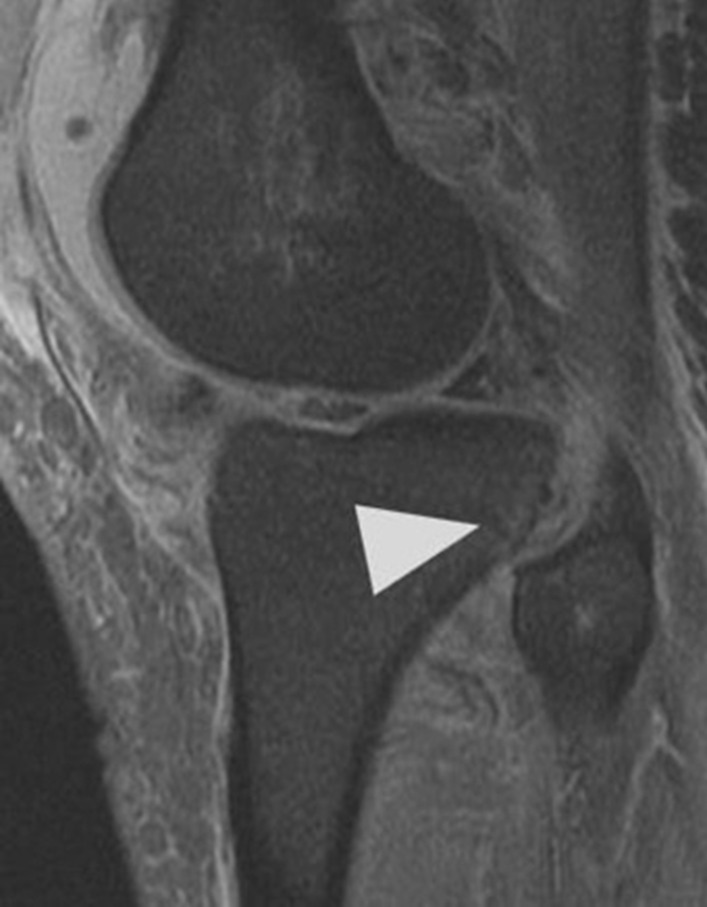
Undisplaced far lateral tibial plateau fracture (indicated by arrowhead), deemed likely to be an avulsion related to lateral ligamentous injury or tibiofibular joint injury

**Fig. 7:**
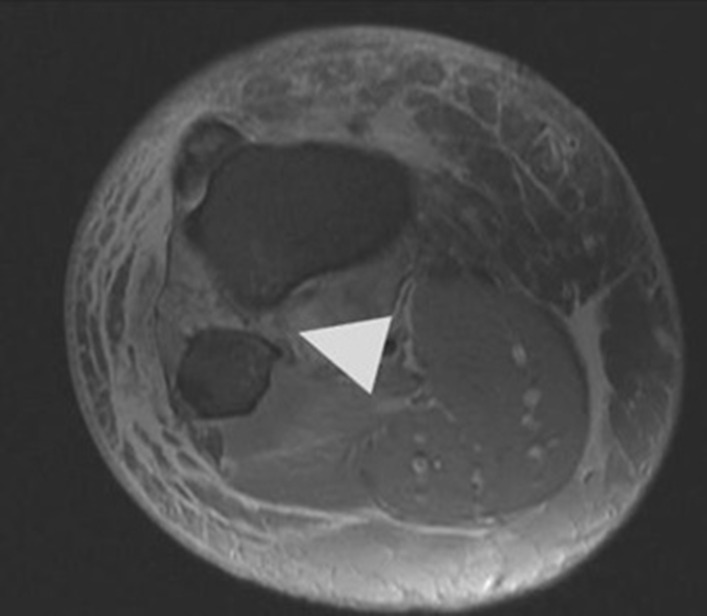
Dislocated proximal tibiofibular joint with the proximal fibula having rotated and separated from the tibia (indicated by arrowhead)

**Fig. 8:**
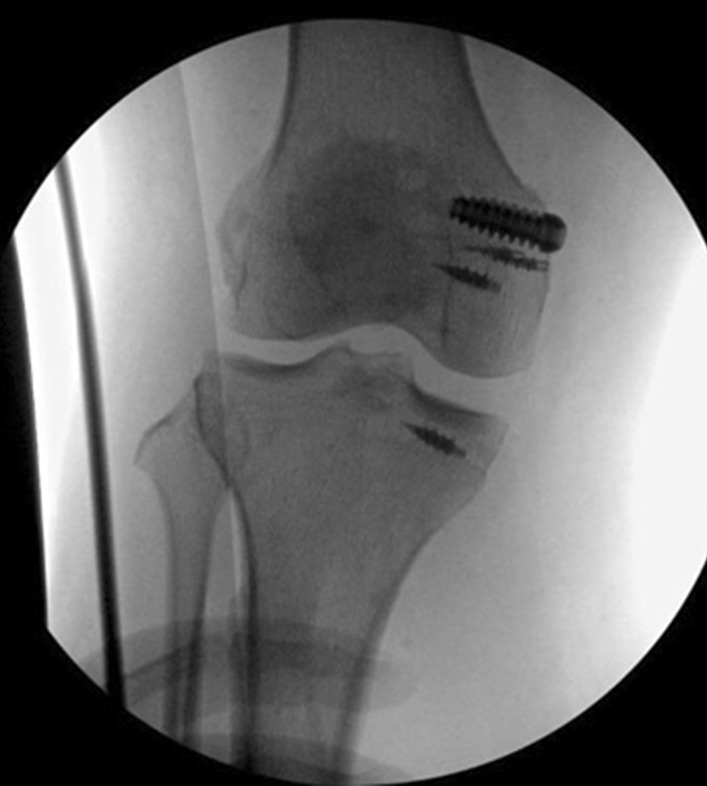
Intraoperative x-rays showing appearance of reconstructed and stable knee following removal of external fixation, anchors *in situ* following medial collateral ligament  repair and medial patellofemoral ligament reconstruction

Initial plain radiographs revealed a recurrence of the dislocation of the patellofemoral joint, with incongruity of the tibiofemoral joint (Figs. 1, 2, and 3). An initial CT in the emergency department showed air within the joint, signifying breach of the capsule from the wound sustained during the injury. At computed tomography (CT) angiogram sequence showed no evidence of vascular injury. CT did however reveal the fibula to be posteriorly dislocated from the tibiofemoral joint. An MRI was arranged as urgent, which confirmed the following:

- 1. A completely torn medial patellofemoral ligament (MPFL) with the lateral patellar dislocation.
- 2. Grade 3 tear of the medial collateral ligament (MCL) at the femoral insertion,
- 3. Grade 2 (partial tear) ACL injury (Fig. 4),
- 4. Displaced bucket handle tear of the medial meniscus (Fig. 5),
- 5. Partially torn (grade 2) posterior cruciate ligament (PCL),
- 6. Completely torn lateral collateral ligament (LCL) and a partial avulsion of the insertion of biceps femoris
- 7. Undisplaced far lateral tibial plateau fracture, deemed likely to be an avulsion related to lateral ligamentous injury or tibiofibular joint injury (Fig. 6)
- 8. Dislocated proximal tibiofibular joint (PTFJ) with the proximal fibula having rotated and separated from the tibia (Fig. 7).

These images were discussed in a multidisciplinary team (MDT) meeting; the consensus was that this combination of injuries represented a transient tibiofemoral dislocation along with the unreduced tibiofibular and patellofemoral dislocations.

### Details of surgical treatment

Despite the delayed presentation to our unit, the patient was treated in accordance with the BOAST 4 guidelines for severe open lower limb injuries. Intraoperatively, wound exploration revealed no signs of early infection, and following a discussion about the various options of treating the wound, it was closed in conjunction with the plastic surgeons. Examination under anesthesia prior to stabilization revealed gross valgus laxity and instability in external rotation. The patient then underwent stabilization of the three joint dislocations:

- 1. Open reduction of the patellofemoral dislocation with reconstruction of the medial patellofemoral ligament with ipsilateral semitendinosus tendon autograft.
- 2. Medial collateral ligament repair with suture anchors at femoral insertion
- 3. Medial meniscal root and body repair.
- 4. Reduction under anesthesia of the proximal tibiofibular joint. After reduction, the joint was stable and did not require additional fixation.
- 5. In view of the open nature of the injury, it was felt that the ACL and PCL partial injuries should be assessed at a later date and not treated with immediate reconstruction due to the risk of septic arthritis. The peripheral tibial plateau avulsion fracture did not require internal fixation.
- 6. A knee-spanning external fixator was constructed to maintain reduction of the tibiofemoral joint and enable inspection of the injured soft tissues.

Following stabilization and repair, the laxity described preoperatively was eliminated. The patient was then treated with intravenous antibiotics for 7 days until it was felt that the wound repair was healing without sign of infection. One week later the wound was inspected again, and the external fixator was removed with a view to reconstruct the cruciate and lateral ligaments. However, examination under anesthesia revealed a stable knee (Fig. 8). Physiotherapy was commenced with the patient in an adjustable range of movement knee brace.

The patient underwent an intensive program of outpatient physiotherapy, hydrotherapy, and hinge knee bracing for 8 weeks. The bracing regime was an initial 0–30° range of movement, followed by 30° increments of flexion range every 2 weeks.

### Outcome and follow-up

**Fig. 9:**
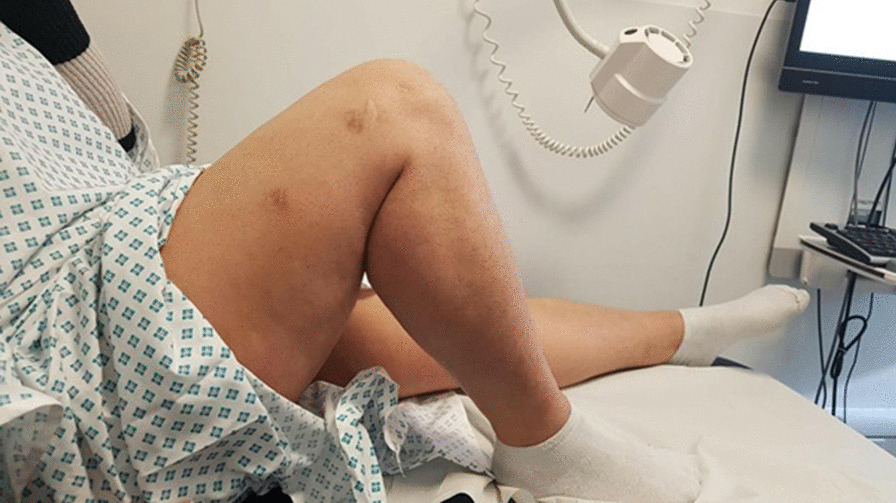
Picture showing flexion of the right knee 1 year postinjury

**Fig. 10:**
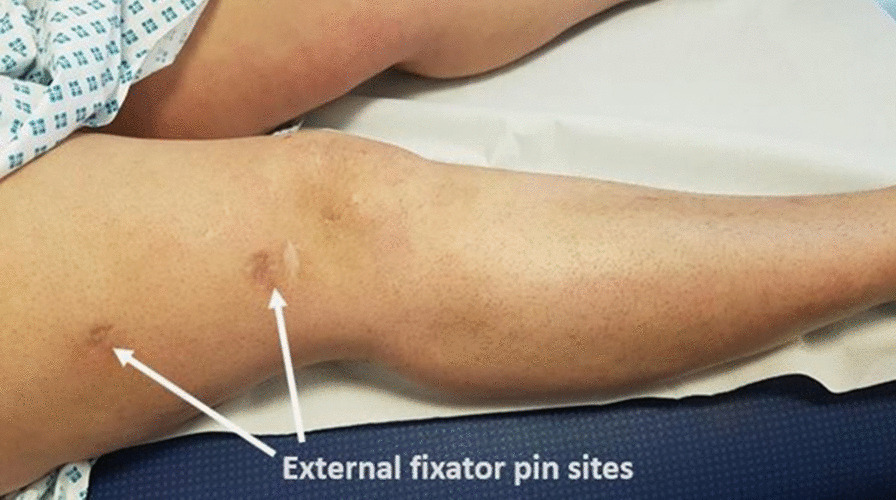
Picture showing extension of the right knee 1 year postinjury. Arrows demarcating previous external fixator pin sites

At 1-year postinjury, the patient was able to walk on the flat without any pain or instability, and could perform most activities of daily living. The patient’s main functional limitation was restriction of flexion that affected going downstairs and kneeling. There was no rest pain or night pain and no reported instability. She had a range of movement from 0° to 120°. The knee was stable to testing of the patellofemoral joint, anterior draw, Lachman’s test, and posterior and posterolateral corner. Although her knee has not completely returned to normality, this represents a satisfactory outcome considering her initial injuries (Figs. 9, 10).

## Discussion

Although there are reports in the literature of tibiofemoral dislocations with either patellofemoral or proximal tibiofibular dislocation, to the authors’ knowledge, this is the only published case of dislocation of all three articulations around the knee joint.

Tibiofemoral dislocation can present after trauma with obvious clinical deformity of this joint. It most often results from damage to at least one cruciate and one collateral ligament. However, there are occasions when the dislocation has occurred at the time of injury, but the joint has reduced spontaneously [6]. The dislocation then has to be inferred from the mechanism of injury, the degree of clinical instability, and the MRI appearances of ligament damage. This certainly applies in our case, with multiple ligaments being injured simultaneously.

In a traumatic presentation of knee dislocation, the main concern is of neurovascular compromise to the lower leg. Fortunately, this patient did not sustain either, perhaps surprising considering the combination of injuries sustained. A large prospective study showed that approximately 20% of patients had a common peroneal nerve (CPN) injury [7] and 5% had an associated vascular injury secondary to traction injuries of the popliteal artery. Vascular injury is more prevalent, especially in anterior dislocations of the knee [8]. It is therefore imperative that the pulses of the foot (dorsalis pedis and posterior tibial) are palpated during assessment. Any suspicion of vascular compromise should be further investigated with angiogram (CT or traditional). In the absence of this modality, ankle/brachial pressure index can be measured, and if below 0.8, should raise suspicion of arterial injury.

Additionally, for tibiofemoral knee dislocations associated with PCL and posterolateral corner injuries, the risk of CPN damage has been quoted to be as high as 45%, with only 21% of CPN injuries recovering useful function [1, 9]. The common peroneal nerve should therefore be thoroughly tested in any patient with suspected knee dislocation, along with the tibial nerve.

Patellofemoral joint dislocations most commonly occur in patients with predisposing factors such as hyperlaxity or patellofemoral dysplasia [2]. In our case, this was probably due to the high energy of medial trauma causing a direct lateral force on the patella, leading to an open wound and complete tear of the medial patellofemoral ligament, which required reconstruction to achieve a stable patella.

Dislocations of the proximal tibiofibular joint (PTFJ) rarely occur in isolation. In a large case series published by Herzog *et al*. 30 patients with PTFJ injuries had either a tibial plateau or diaphyseal tibial fracture [5]. In the referenced study, these injuries were stabilized with screw fixation. In contrast, our patient did not require any operative stabilization of this joint as it was stable after reduction. Additionally, we were mindful of a potential injury to the common peroneal nerve but there was no evidence of this, either on examination or intraoperatively. In this case, the tibiofibular dislocation was significant in the context of an avulsion of the biceps femoris and the LCL rupture, showing that a tibiofibular dislocation can potentially be a sign of an associated PLC injury.

The treatment of multiligamentous knee injuries can be conservative or operative, depending on the patient’s comorbidities, structures damaged, and the resulting instability of the knee. However, in the case of high-energy trauma and three ligament damage, surgical reconstruction or repair of the ligaments is required for most patients. Several reports have suggested that tibiofemoral dislocations can be associated with dislocations of the other joints of the knee [3]. A series published in 2001 studied four cases of tibiofemoral dislocations associated with patellofemoral dislocation, quoting that 16% of tibiofemoral dislocations had a simultaneous patellar dislocation [4]. The proximal tibiofibular joint has also been implicated in tibiofemoral dislocations. CPN injury is the concern with this injury due to the nerve coursing around the neck of the fibula. In 2015, a study of 30 patients with CPN injuries secondary to proximal tibiofibular dislocations suggested that they have similar outcomes to tibiofemoral dislocations. However, the study was primarily looking at CPN injuries associated with tibial fractures [5].

Tibiofemoral knee dislocations can be classified using the Schenck classification (Table 1).

**Table 1:** Schenck classification of tibiofemoral knee dislocations

Schenck classification	Injured structures
I	Single cruciate and collaterals
II	ACL + PCL and intact collaterals
III M	ACL + PCL + MCL
III L	ACL + PCL + LCL
IV	ACL + PCL + MCL + LCL
V	Dislocation + fracture

Considering that our patient had a lateral tibial plateau fracture, her injuries would have constituted a Schenck V injury. Schenck V injuries can be further classified from V1 to V4 [10], (Table 2).

**Table 2:** Schenck V sub classification of tibiofemoral knee dislocations

Class V (fracture/dislocation) subdivision	Injured structures
V1	ACL or PCL
V2	ACL + PCL
V3 M	ACL + PCL + MCL
V3 L	ACL + PCL + LCL/PLC
V4	ACL + PCL + MCL + LCL/PLC

Additionally, our patient sustained partial injuries of both cruciate ligaments and complete ruptures of both collateral ligaments, which is an almost complete loss of integrity of the tibiofemoral joint. This is a Schenck class V4 fracture dislocation, and underlines the severity of the injuries in this report. The preferred treatment here would have been to reconstruct all four ligaments and the patellofemoral ligament at one operation, but it was felt that a two-stage reconstruction would be more prudent in view of the large open medial wound and delayed presentation for definitive surgery. It was fortuitous that following removal of the external fixator, the tibiofemoral joint was stable and did not require further soft tissue reconstruction.

The use of a spanning external fixator to stabilize the knee is a safe option in treating multiligamentous soft tissue knee injuries in the presence of open injury. For our patient, the external fixator not only allowed us to protect the ligamentous reconstruction, it also gave us the ability to monitor the wound and soft tissue status of the knee. This was vital as she presented with an open wound communicating with the knee joint. We monitored the knee for persistent ACL, PCL, or lateral/posterolateral laxity throughout rehabilitation, in case subtle laxity requiring reconstruction became apparent. However, the knee remained stable in all directions and no further procedure was required.

The latest study of long-term outcomes of knee dislocations was performed by Fanelli *et al*. in 2014 [11], which was a case series of 44 knee dislocations with an average follow-up of 10 years. The average patient age of the study was 31 years, and the study showed that most patients achieved a range of movement of 0–120° and had achieved static and functional stability, allowing the return to physically demanding work and recreational activities. However, up to nearly 25% of cases developed marked osteoarthritis by the end of the follow-up period. For our case, a 1 year follow-up is by no means enough to comment on long-term outcomes. But with other studies reporting rates of osteoarthritis of up to 80%, this is a relevant discussion point as our patient was 40 years old at the time of injury and independently mobile.

## Conclusion

Dislocation of the knee is an uncommon but life-changing and potentially limb-threatening injury. It should always be suspected in trauma patients who present with multiligamentous knee injuries. The main concern is of neurovascular compromise to the lower leg, namely, the popliteal artery and common peroneal nerve. These structures should be clinically examined at presentation, with angiography performed if arterial damage is suspected.

The treatment of multiligamentous knee injuries for most patients is surgical treatment with physiotherapy and adequate stabilization of the knee joint. Close monitoring of progress of the knee in terms of persistent laxity, range of movement, and functional status is required for at least 1-year postinjury. Current evidence suggests that, despite good functional outcomes for knee dislocations in the short term, the prevalence of posttraumatic osteoarthritis is high in the long term.

## Data Availability

Not applicable.
